# Are different medical school admission tests associated with the outcomes of a simulation-based OSCE?

**DOI:** 10.1186/s12909-021-02703-x

**Published:** 2021-05-07

**Authors:** Lisa Bußenius, Sigrid Harendza

**Affiliations:** 1 Department of Biochemistry and Molecular Cell Biology, Center for Experimental Medicine, University Medical Center Hamburg-Eppendorf, Hamburg, Germany; 2 III. Department of Internal Medicine, University Medical Center Hamburg-Eppendorf, Hamburg, Germany

**Keywords:** Communication, Medical school admission, Medical school selection, Medical knowledge, Multiple mini-interviews, Objective structured clinical examination, Undergraduate medical education

## Abstract

**Background:**

Medical school admission procedures have the common goal to select applicants with the greatest potential of becoming successful physicians. Hamburg Medical Faculty selects medical students by grade point average (GPA) and employs a two-step selection process of a natural sciences test (HAM-Nat), in some cases followed by multiple mini-interviews (HAM-Int). Multiple mini-interviews can predict non-cognitive outcomes, while GPA has predictive validity for cognitive outcomes. The aim of our study was to explore communication skills and clinical knowledge of advanced medical students according to their respective admission procedure.

**Methods:**

In July 2019, 146 students grouped according to their admission procedure into GPA-only (19.2 %), HAM-Nat (33.6 %), HAM-Int (30.8 %), and Waiting List (16.4 %) participated in four OSCE stations which equally assessed students’ communication skills (OSCE part 1) and clinical knowledge (OSCE part 2) in simulated patient encounters, rated by physicians with checklists. Additionally, psychosocial assessors ranked communication skills with a global rating scale (GR). The students also participated in a multiple choice (MC) exam testing clinical knowledge. Kruskal-Wallis analyses of variance of test performance and Spearman correlation of instruments were calculated.

**Results:**

Students from the Waiting List group performed significantly worse on the MC exam compared to GPA-only and HAM-Int (adjusted *p* = .029 and 0.018, respectively). No significant differences were found between the admission groups with respect to communication skills. Global Rating and OSCE part 1 (communication) correlated significantly (*ρ* = 0.228, *p* = .006) as did OSCE part 2 (clinical knowledge) and MC exam (*ρ* = 0.242, *p* = .003), indicating criterion validity. Constructs did not overlap, indicating divergent validity.

**Conclusions:**

Advanced medical students selected for undergraduate studies by multiple mini-interviews assessing psychosocial skills showed similar communication skills compared to students admitted to medical school by other entryways. It is unclear whether these similarities are due to an effective undergraduate longitudinal communication curriculum. Assessing baseline communication skills of all medical students at entry-level may aid with this question.

**Supplementary Information:**

The online version contains supplementary material available at 10.1186/s12909-021-02703-x.

## Background

Medical school selection processes show a great variety [1]. Choosing the ideal candidates for undergraduate medical studies is often a balance between the question which students will most likely become successful future doctors and the feasibility of different admission procedures [2, 3]. Among the most practiced selection methods, high school records, multiple mini-interviews (MMIs), aptitude tests, situational judgment tests (SJTs), and selection centres (SCs) are considered fairer than unstructured interviews, personal statements, or references [4]. As they are adaptable to the specific medical schools’ values and requirements [5], MMIs differ on the constructs they intend to measure, but e.g. communication skills, integrity, empathy, or ethical decision making are frequently assessed domains [6]. While high school grade-point average (GPA) seems to have predictive validity for cognitive outcomes of undergraduate medical education [7, 8], a systematic review showed that both MMIs and SJTs can predict non-cognitive outcomes such as personal attributes, e.g. empathy and integrity [4], which are desirable traits for successful medical graduates besides medical knowledge [9].

In Germany, all applications to medical school are processed by a central administration office. In 2020, 49,885 high school graduates applied for the 9660 places available for undergraduate medical training in total [10], making it one of the most competitive study programmes in the country. Applicants are ranked according to their GPA and waiting time (in semesters). Each group receives approximately 20 % of the available spots in undergraduate medical studies. The remaining 60 % are appointed in a decentralized fashion by the individual medical schools. Each academic year, Hamburg University accepts about 380 to 400 students depending on capacity. Since 2010, the Hamburg Medical Faculty invites approximately 1,000 to 1,300 applicants based on a cut-off GPA to sit a natural sciences test (HAM-Nat) and accepts the best 100 [11]. After this process, the applicants ranked from 101 to 300 are invited to participate in a validated MMI (HAM-Int), which measures psychosocial competencies such as empathy, communication skills, and self-regulation [12, 13]. Thereafter, the 100 best applicants of that cohort get admitted to undergraduate medical studies.

To account for both cognitive and non-cognitive outcomes, measurable and valid assessment formats have to be determined for undergraduate medical education. Among other assessment methods, multiple-choice (MC) exams are typically used to assess cognitive knowledge in medical education [14] while objective structured clinical examinations (OSCEs) facilitate assessment of practical skills and clinical competence in the health professions [15, 16]. OSCEs can also be employed to measure communication skills [17] which are essential for practicing physicians [18–20]. We hypothesise that MC exam results and OSCE results of advanced students are suitable to measure outcome performance and therefore could indicate predictive validity of admission tools. In this study, we sought to explore differences in both cognitive and non-cognitive outcomes between students admitted to undergraduate medical studies according to their admission procedure. We suspect that students whose GPA or knowledge of natural sciences was of relevance for their admission show better performance in the MC exam. Furthermore, we hypothesise that students admitted for their psychosocial competencies perform better in the communication parts of OSCEs.

## Methods

### Study design and participants

Undergraduate medical studies in Germany last six years with the final year being spent entirely in clinical or outpatient rotations [21]. The undergraduate medical curriculum at the Medical Faculty Hamburg, called *Hamburg integrated Medical Degree Program* (iMED), consists of 19 modules in the first five years with integrated pre-clinical and clinical content of increasing complexity [22]. The module focused on in this study comprises the topics “abdomen, retroperitoneum, endocrine system, and metabolism” on the highest level of complexity in year four to five [23]. Passing this module requires two exams: (1) an OSCE (7 stations, six minutes each) constituting 35 % of the final module grade, and (2) a written MC test (65 questions covering 13 subjects) constituting 65 % of the final module grade. We redesigned four OSCE stations (general surgery, gastroenterology, nephrology, and urology) to particularly assess students’ communication skills and clinical knowledge in simulated physician-patient encounters. For every station, we developed two different case scenarios of equal difficulty that changed during the testing day to reduce opportunities for cheating. In each of the four new stations, a standardized patient (trained actor) as well as two examiners were present. The first examiner was a physician of the respective station’s medical specialty who rated the students’ academic knowledge with a clinical checklist regarding aspects of communication (history taking), i.e. OSCE part 1, and clinical knowledge (differential diagnoses and treatment decisions), i.e. OSCE part 2. The second examiner was a psychologist or sociologist who rated the students’ communication skills with the Global Rating Scale (GR) [24–26]. Only the first examiner’s ratings on the clinical checklist were relevant for the students’ OSCE grade. In each station, the student was supposed to communicate with the standardized patients without knowing the nature of the patients’ diseases. Both examiners were passive observers. All ratings as well as student identification were logged via Apple iPads Air A1474 running the tOSCE application (Umbrella Consortium for Assessment Networks, Heidelberg, Germany).

The OSCE took place on two consecutive days in July 2019 to accommodate all students of the module. Ten trained physician examiners (two general surgeons, four gastroenterologists, two nephrologists, and two urologists) and seven trained communication assessors (five psychologists and two sociologists), as well as eight actors performing as standardized patients (one per case scenario) took part in the assessment. On day one, 97 students, and on day two, 89 students were examined. Additionally, all students participated in the MC exam three or four days later, respectively.

Applicants chosen by Hamburg Medical Faculty’s selection tests signed informed consent prior to the admission procedure and the Ethics Committee of the Hamburg Chamber of Physicians approved of this study (WF-047/16) under the condition that data were anonymized. Students were informed about the additional ratings prior to the OSCE and were able to deny these if they wished. This study was part of our evidence-based development program of rating instruments taking place during regular exams as part of Hamburg University’s quality assurance policy. All data were anonymized and consolidated by a third party neither involved in examining nor data analysis.

### Instruments

#### OSCE checklists

Each OSCE checklist was tailored specifically for a respective station’s content and case, diagnoses being derived from the learning objectives of this module. Every checklist rewarded ten points for communication during history taking (OSCE part 1) and another ten points for clinical knowledge through explaining differential diagnoses and treatment (OSCE part 2). Checklists cannot be disclosed further due to their utilization in official university examinations.

#### MC exam

The MC exam consisted of 65 questions from 13 medical disciplines. Each question was followed by four answer options with one single correct answer. For this study, we extracted the 29 questions of the relevant disciplines (general surgery, gastroenterology, nephrology, and urology).

#### Global Rating Scale (GR)

The global rating scale was developed to assess communication skills in OSCEs and complement traditional checklists [24, 25]. It was later adapted to the German-speaking context [26]. It consists of four scales: Response to patient’s feelings and needs (empathy), degree of coherence, verbal expression, and nonverbal expression. Each scale is rated on a five-point Likert scale (1: poor performance to 5: perfect performance in the respective domain) with verbal anchors for each pole to facilitate decision-making. We adhered to Scheffer et al.’s translation but inverted their scale back to Hodges et al.’s original scale to make scores in the clinical checklist and GR compatible. The final questionnaire used in this study is provided as supplementary material (Additional file 1).

### Data processing

Data were analysed using IBM SPSS Statistics for Windows, version 26 (IBM Corp., Armonk, N.Y., USA) with a general alpha-level of 0.05. We identified admission quotas from 2015 and assigned students to four groups according to their final acceptance to medical school: GPA-only, HAM-Nat, HAM-Int, and Waiting List. GR subscales, both parts of the OSCE checklists, and the 29 relevant MC questions were individually summed up and the final scores were converted to percentages to enable direct comparison. We checked the normality assumptions of these scores with Kolmogorov-Smirnov-tests. As data were non-parametric, group differences between the four student groups (independent variables) were calculated with Kruskal-Wallis-tests with the dependent variables of age and GPA. We also analysed group differences of exam performance with the four dependent variables of MC, OSCE part 1, OSCE part 2, and GR with independent Kruskal-Wallis-tests and, where applicable, post-hoc Conover-Iman-tests [27], corrected for multiple testing according to Benjamini & Hochberg [28], using the R-package conover.test [29] in R version 4.0.3 (R Core Team, Vienna, Austria). Associations between the four variables were assessed with Spearman correlations. Significant correlations were further examined for individual admission groups.

## Results

Of the 186 students who participated in the OSCE, the admissions quota could not be identified for 40 students. We assigned the remaining 146 students (63 % female) to the four admission groups (Table 1; GPA-only: 19.2 %, HAM-Nat: 33.6 %, HAM-Int: 30.8 %, Waiting List: 16.4 %). The distribution of admission groups and gender was skewed (χ^2^(3) = 9.61, *p* = .022). There were significant differences in the four groups regarding age (χ^2^(3) = 60.35, *p* < .001) and GPA (χ^2^(3) = 105.14, *p* < .001). Students admitted by waiting list were significantly older than students from all other admission groups. Students admitted by GPA only had significantly better GPA scores and students admitted by waiting list had significantly worse GPA scores than all other groups. Students admitted through the HAM-Nat had significantly better GPA scores than those admitted by the HAM-Int.

**Table 1:** Participants’ characteristics

		Gender	Age (in years)	GPA
**Admission Quota**	**N (%)**	**f / m**	**M ± SD**	**M ± SD**
GPA-only	28 (19.2 %)	22 / 6	24.43 ± 2.64	1.01 ± 0.04***
HAM-Nat	49 (33.6 %)	23 / 26	23.96 ± 1.62	1.40 ± 0.22*
HAM-Int	45 (30.8 %)	32 / 13	23.93 ± 1.47	1.51 ± 0.20*
Waiting List	24 (16.4 %)	15 / 9	32.83 ± 4.58***	2.85 ± 0.41***

German GPA scores are negatively polarized, 1.0 being the best, 4.0 being the lowest grade

*f *female, *m *male

Age: *** *p* < .001: Waiting List versus GPA-only, HAM-Nat, and HAM-Int

GPA: *** *p* < .001: Waiting List versus GPA-only, HAM-Nat, and HAM-Int; GPA-only versus HAM-Nat, HAM-Int and Waiting List; * *p* = .002: HAM-Nat vs. HAM-Int

**Fig. 1:**
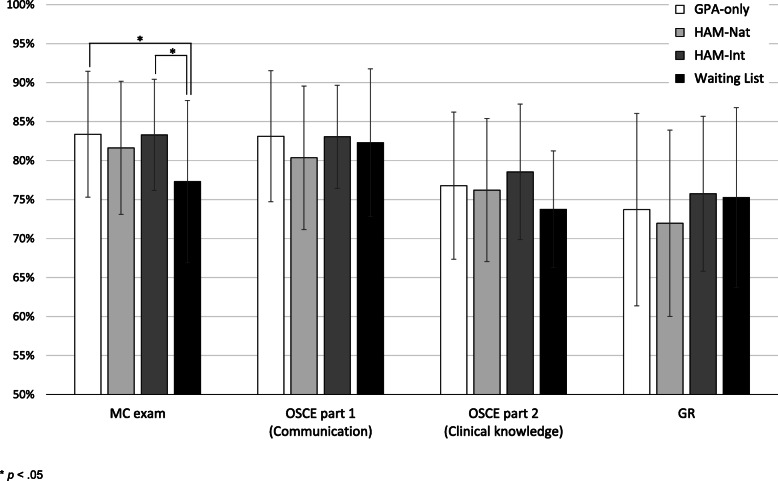
Mean performance in different exam parts

Figure 1 shows the outcome of all admission groups for the four different parts of the exam (MC exam, OSCE part 1 (communication), OSCE part 2 (clinical knowledge), and GR). The MC exam revealed significant differences (χ^2^(3) = 8.09, *p* = .044): Students from the admission groups GPA-only (*M* = 83.37 ± 8.08 %) and HAM-Int (*M* = 83.30 ± 7.12 %) performed significantly better on the MC exam than students from the admission group Waiting List (*M* = 77.30 ± 10.42 %; adjusted *p* = .029 and .018, respectively). No significant differences were found between the admission groups for OSCE part 1 (communication), OSCE part 2 (clinical knowledge), and GR.

**Fig. 2:**
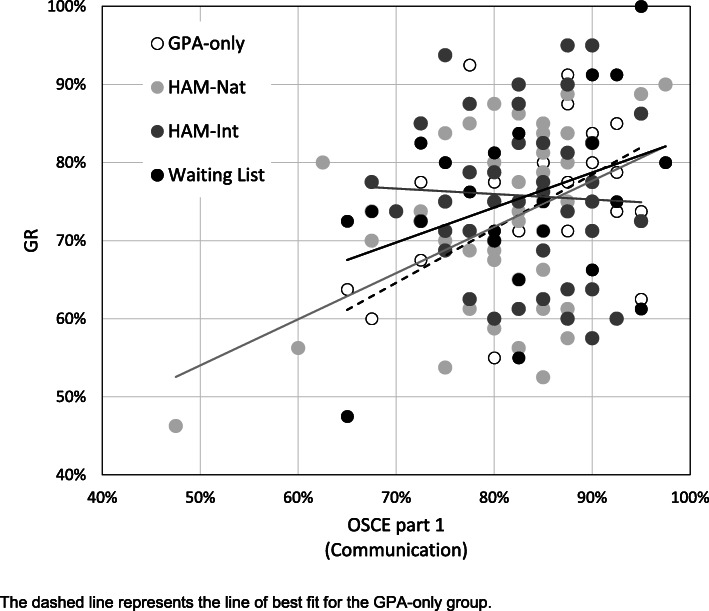
Correlation of GR with OSCE part 1 (Communication)

**Fig. 3:**
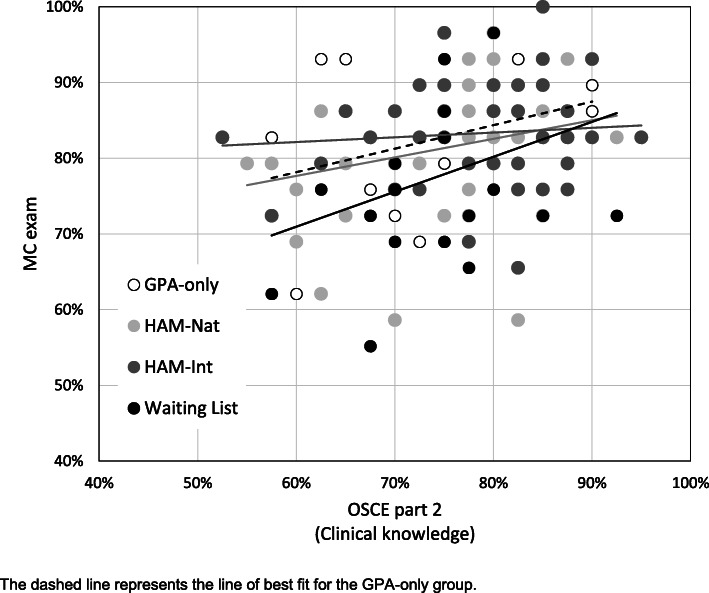
Correlation of MC exam with OSCE part 2 (Clinical knowledge)

In general, GR and OSCE part 1 (communication) correlated significantly (ρ = .228, *p* = .006): Fig. 2 reveals a significant correlation for the HAM-Nat group (ρ = .297, *p* = .038), but not for the GPA-only, Waiting List or HAM-Int group. Overall, there was a significant correlation of MC exam and OSCE part 2 (ρ = .242, *p* = .003) while no significant correlations were found for the individual admission groups (Fig. 3). OSCE part 1 did not correlate with either measure of clinical knowledge (OSCE part 2: ρ = .029, *p* = .690; MC: ρ = .066, *p* = .372) and neither did GR (OSCE part 2: ρ = .081, *p* = .271; MC: ρ = .129, *p* = .079).

## Discussion

In our study, we analysed both cognitive and non-cognitive assessment outcomes with respect to the students’ entryways to undergraduate medical studies in order to examine significant relationships between the performance of advanced undergraduate medical students in relation to their respective medical school admission procedure. Outcomes were defined as scores on a simulation-based OSCE, a communication GR, and an MC exam in fourth year medical students.

Students admitted through a waiting list quota performed significantly worse on the MC exam than those admitted for their excellent GPA or HAM-Int scores. This is in line with earlier findings that students admitted via a waiting list show inferior performances in knowledge tests [30–32]. This is a problematic finding, as knowledge and its availability are a critical foundation for clinical reasoning [33], a key element of physicians’ competences. The waiting list students’ lower test scores might be due to age-related factors and generally lower GPA in this group of students, associated with lower study success [32]. Additionally, this group shows dropout rates up to 20 % [32].

We did not find significant differences between the different admission groups regarding OSCE part 1 (communication) scores or GR ratings. This finding was surprising as we had hypothesised that students selected through multiple mini-interviews (HAM-Int) would outperform all other groups in regards to communication skills, as the purpose of the MMI was to select candidates showing excellent psychosocial competence. However, communication training plays an important part in our undergraduate medical curriculum with a longitudinal training course accompanying the students through the entire curriculum [22]. Research shows ample evidence for the effectiveness of medical students’ communication skills training [34–36]. Therefore, four years of continuous communication training might have efficiently brought students’ communicative competence to a similar level regardless of their communication skills at the time of medical school admission. Furthermore, the predictive power of admission tools is limited – the quality and validity of the assessment of psychosocial competences varies between tests, and cognitive and non-cognitive performance are not mutually exclusive [37]. The instruments for measuring communication skills (GR) and history taking (OSCE part 1) correlated weakly to moderately. This may indicate that there is more to history taking than simply asking the right questions, supporting a process-based communication model of the medical interview as opposed to a content-based approach [38], acknowledging the limited explained variance of 5.2 %. The correlation of GR and OSCE part 1 is particularly noticeable for the HAM-Nat admission group, which is one of the groups usually showing best exam performance [30]. Criterion validity can also be shown for the results of the MC exam and knowledge shown in OSCE part 2 (clinical knowledge). The weak to moderate correlation could be explained by context specificity [39]. The instruments used for communication skills as well as medical knowledge did not correlate which indicates divergent validity.

This study had certain limitations. The groups are not evenly distributed regarding age and gender. The waiting list group is significantly older, hence introducing possible bias. However, the distribution of quotas was not significantly different from the original admissions quota and could thus be considered representative of the original student population. Furthermore, the sample size in each group is small and the entryways to medical school could not be identified for 40 students possibly reducing power. While the performance measures correlate consistently with construct assumptions, all groups scored substantially over the pass mark of 60 % in both OSCE and MC exam. Differentiation between groups may improve by more difficult or complex testing thus reducing the ceiling effect [40]. Additionally, medical school admission procedures vary greatly between universities nationally and internationally. Thus, our results cannot easily be generalized. However, in the general debate concerning medical school admission, international processes seem to move in the same direction. In the USA, most admission officers rely on two-step or three-step processes with interviews in addition to MCAT scores and undergraduate GPA [41], while the UK currently relies on three measures including academic achievements, aptitude tests such as the UKCAT, and interviews [42]. A strength of our approach is the introduction of the GR as an additional rating instrument for communication, which increased criterion validity for communication in addition to OSCE part 1 (communication). The learning module chosen for our study was of the highest difficulty level in the curriculum with respect to both in-depth knowledge as well as acquired communication competence. Therefore, it seemed ideal to correlate these outcomes with the admission tests measuring these two components.

An optimal approach to compare admission groups would include entry-level performance scores of all students on psychosocial skills regardless of their actual admission path. This would allow for direct comparison of subgroups and larger scale regression models to better evaluate predictive validity of admission tools.

## Conclusions

Students who were admitted to medical school through multiple mini-interviews focusing on psychosocial skills, did not outperform medical students who were admitted by their high school grade, a natural sciences test or a waiting list when tested specifically for communication skills. However, it is unclear if this is due to the locally well-established longitudinal undergraduate communication curriculum. Assessing baseline communication skills at entry-level for all medical schools would allow for better prediction of these skills throughout undergraduate medical studies.

## Supplementary Information

**Additional file 1.** Global Rating Scale for the assessment of communication skills

## Data Availability

All data and materials are available from the corresponding author upon request.

## Abbreviations

GPA: Grade-point average
GR: Global Rating scale
HAM-Int: Hamburg Assessment Test for Medicine–Interview
HAM-Nat: Hamburg Assessment Test for Medicine–Natural Sciences
iMED: Hamburg integrated Medical Degree Program
MC: Multiple-choice
MMI: Multiple mini-interview
OSCE: Objective structured clinical examination
SJT: Situational judgment test
SC: Selection center
