# Supplementary material for: Are different medical school admission tests associated with the outcomes of a simulation-based OSCE?
Source: BMC Med Educ. 2021 May 7;21:263. doi: 10.1186/s12909-021-02703-x (PMC8103591; doi:10.1186/s12909-021-02703-x)
Supplement: Supplementary file 1 — Additional file 1. Global Rating Scale for the assessment of communication skills [file 12909_2021_2703_MOESM1_ESM.docx]

**Global** **Rating Scale for the assessment of communication skills**

| **Response to patient emotions and needs (empathy):** | | |
| --- | --- | --- |
| Does not respond to obvious patient cues (verbal and nonverbal)  **and ⁄ or**  responds inappropriately. | 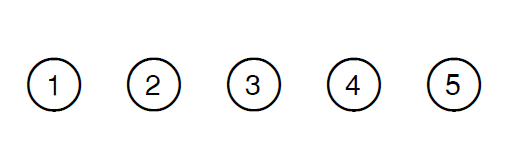 | Responds continuously in a sympathetic way to (verbal and nonverbal) patient cues and needs  **and / or**  responds appropriately. |
| **Degree of interview coherence:** | | |
| No recognisable structure to the conversation; coherence of the conversation is missing or the patient determines the structure of the interview. | 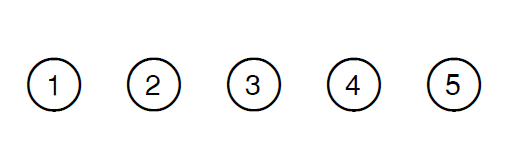 | Excellent structure of the conversation, demonstrating cohesiveness and leadership in the interview. |
| **Verbal expression:** | | |
| Communicates in a way that complicates or hinders patient comprehension  **and / or**  communicates inappropriately with the patient  (regarding wording, semantics, grammar, tone, volume, voice modulation, and pronunciation). | 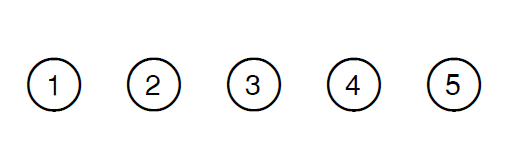 | Communicates in a way that facilitates patient understanding  **and / or**  communicates appropriately with the patient (regarding wording, semantics, grammar, tone, volume, voice modulation, and pronunciation). |
| **Nonverbal expression:** | | |
| Fails to engage the patient by nonverbal expressions and frustrates the patient  **and ⁄ or**  raises patient anger (e.g. by eye-contact, gesture, posture, pauses). | 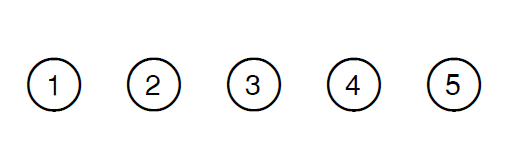 | Engages the patient consistently by nonverbal expressions  **and / or**  motivates the patient to engage in the conversation (e.g. by eye-contact, gesture, posture, pauses) |

This is a translated version of the rating scale for the assessment of communication skills [23], which is based on an analytic global rating form [22]. The 5-point scale adheres to the original version [22].
